# Impairment of starch biosynthesis results in elevated oxidative stress and autophagy activity in *Chlamydomonas reinhardtii*

**DOI:** 10.1038/s41598-019-46313-6

**Published:** 2019-07-08

**Authors:** Quynh-Giao Tran, Kichul Cho, Su-Bin Park, Urim Kim, Yong Jae Lee, Hee-Sik Kim

**Affiliations:** 1 Cell Factory Research Center, Korea Research Institute of Bioscience and Biotechnology (KRIBB), Daejeon, 34141 Republic of Korea; 2 Department of Environmental Biotechnology, KRIBB school of Biotechnology, Korea University of Science & Technology (UST), Daejeon, 34113 Republic of Korea; 3 Environmental Safety Group, Korea Institute of Science and Technology (KIST) Europe, Campus E 7.1, Saarbrücken, 66123 Germany

**Keywords:** Biophysical chemistry, Macroautophagy

## Abstract

Autophagy is a self-degradation system wherein cellular materials are recycled. Although autophagy has been extensively studied in yeast and mammalian systems, integrated stress responses in microalgae remain poorly understood. Accordingly, we carried out a comparative study on the oxidative stress responses of *Chlamydomonas reinhardtii* wild-type and a starchless (*sta6*) mutant previously shown to accumulate high lipid content under adverse conditions. To our surprise, the *sta6* mutant exhibited significantly higher levels of lipid peroxidation in the same growth conditions compared to controls. The *sta6* mutant was more sensitive to oxidative stress induced by H_2_O_2_, whereas the wild-type was relatively more resistant. In addition, significantly up-regulated autophagy-related factors including *ATG1*, *ATG101*, and *ATG8* were maintained in the *sta6* mutant regardless of nitrogen availability. Also, the *sta6* mutant exhibited relatively higher ATG8 protein level compared to wild-type under non-stress condition, and quickly reached a saturation point of autophagy when H_2_O_2_ was applied. Our results indicate that, in addition to the impact of carbon allocation, the increased lipid phenotype of the *sta6* mutant may result from alterations in the cellular oxidative state, which in turn activates autophagy to clean up oxidatively damaged components and fuel lipid production.

## Introduction

With the growing demand for environmentally friendly sustainable energy alongside an increasing world population, the subject of algal biomass production has attracted much attention since microalgae can convert atmospheric CO_2_ and water pollutants into diverse value-added products including biofuel and other bioactive compounds^1,2^. Among various compounds, algal lipid, which is mainly composed of triacylglycerol (TAG), is considered a promising resource for biodiesel production^2^. The extracted algal lipid contents can be converted into biodiesel and glycerol via a transesterification reaction with alcohols and catalysts^2^. Furthermore, the overexploitation of fish oil to produce bioactive fatty acids including eicosapentaenoic acid (EPA) and docosahexaenoic acid (DHA) can be solved by using microalgae as alternative resources^1^. The advantages of lipid production using microalgae over terrestrial plants have already been well discussed in previous studies. Microalgae can be grown in wastewater and are able to accumulate higher lipid content with a faster growth rate than common terrestrial plants. In addition, the cultivation of microalgae does not require large areas of land, and they do not compete with general human food resources^2,3^. However, cost-effective lipid production from microalgae remains in its infancy owing to poorly developed mass cultivation techniques and the low lipid productivity of target microalgal species. In order to promote lipid production from microalgae, many technological approaches including genetic engineering, stress regulation, and modification of cultivation apparatus have been proposed^4–7^. One of these approaches, the construction of more efficient algal mutants using genetic engineering, is considered a promising and important subject in the microalgae-to-bioproducts process^8^.

Changes made to cellular metabolic pathways via genetic modification in microalgae can enhance or reduce specific metabolite production, thereby artificially promoting lipid production. Among several strategies, enhancing algal lipid production by altering the starch biosynthesis pathway has been the most widely used technique in diverse microalgal candidates. According to de Jaeger *et al*., genomic DNA of the freshwater microalga *Scenedesmus obliquus* was randomly modified by UV light irradiation to obtain a starchless mutant, which exhibited enhanced lipid accumulation compared to the wild-type under nitrogen-depleted conditions^9^. In addition, Li *et al*. also reported that the starchless *C. reinhardtii* mutant BAFJ5 (referred to as *sta6*), which is defective in ADP-glucose pyrophosphorylase, exhibited approximately 10-fold higher TAG accumulation than the wild-type^10^. Considerable efforts have been made to address the allocation of carbon toward lipids in mutants by modifying their starch biosynthesis metabolism^9,10^. However, little attention has been paid to the regulation of autophagy and the stress response in these genetically modified algal strains.

It has been previously reported that most microalgal species can accumulate increased lipid content under stress conditions, such as nitrogen starvation, oxidative stress and high salinity^11^. For instance, Yilancioglu *et al*. showed a halophilic microalga *Dunaliella salina* accumulated cellular lipid content and exhibited extensive lipid peroxidation under nitrogen deficiency condition, indicating high level of oxidative stress^12^. The study also confirmed that hydrogen peroxide, an oxidative stress inducer, significantly induced algal lipid accumulation^12^. Additionally, H_2_O_2_ treatment and UV irradiation has been shown to enhance lipid production in *Scenedesmus* sp.^13^. These studies suggest that lipid accumulation is highly influenced by oxidative stress in microalgae. Although the exact mechanism of this widespread phenomenon has not yet been elucidated, lipid accumulation has been linked to an increase in reactive oxygen species (ROS) along with the degradation of cellular protein components in various algal species^11,12,14^. On the other hand, ROS-mediated stress responses and protein turnover are closely related to the autophagy mechanism^15–17^. Autophagy is a highly conserved catabolic process that regulates cellular component degradation and recycling in a wide range of eukaryotic organisms^16^. Since it is considered that autophagy plays a significant role in the stress response of eukaryotic cells, the study of the relationship between ROS-mediated stress and autophagy induction is essential^18,19^. Recently, Couso *et al*. demonstrated that maintaining normal autophagic flux is essential for lipid production in *C. reinhardtii*^15^. Based on previous studies, it is hypothesized that cellular stress levels and the subsequent regulation of autophagy may also be correlated with an enhanced TAG accumulation in starchless mutants. Since this has not yet been concretely investigated, in the present study, we examined the nitrogen starvation-induced oxidative stress response and autophagy regulation in the starchless *sta6* mutant of *C. reinhardtii*.

## Results and Discussion

### Confirmation of experimental algal strains

**Figure 1:**
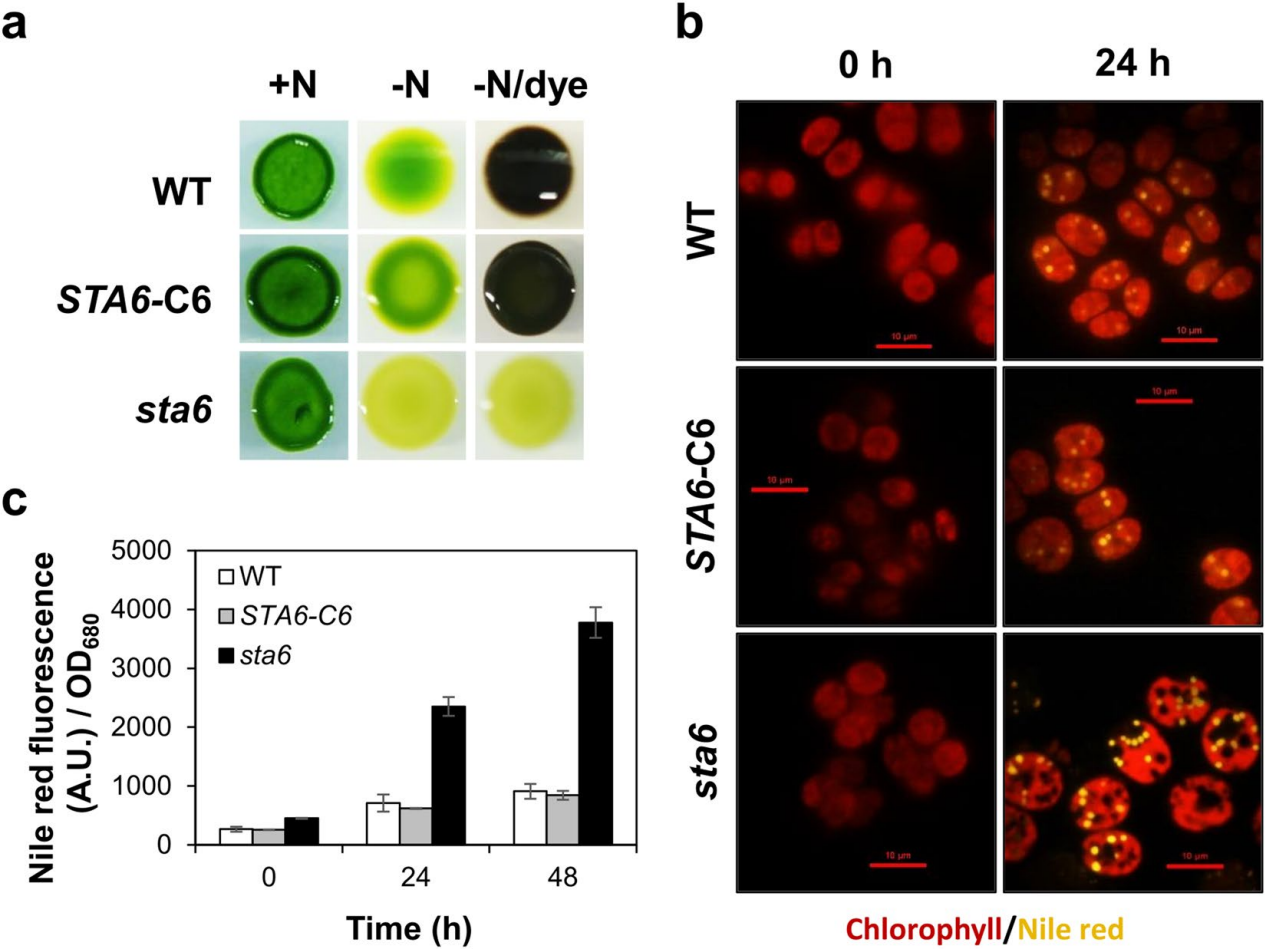
Confirmation of experimental algal strains. (**a**) Wild-type (WT), complemented *STA6* mutant (*STA6-*C6) and starchless mutant (*sta6*) were plated on N-depleted TAP medium for 7 days and the plates were incubated with iodine vapor. WT and *STA6-*C6 colonies were stained to dark brown, indicating the presence of starch. In contrast, the *sta6* mutant was an opaque yellowish green, meaning that they failed to synthesize starch molecules. (**b**) Observation of lipid accumulation in *Chlamydomonas* cells under nitrogen starvation. Lipid droplets stained with Nile Red (yellow) and algal auto-fluorescence (red) are shown. Scale bar = 10 µm. (**c**) Relative Nile Red fluorescence normalized to optical density (OD_680_) of algal cells during a time course of nitrogen starvation. Error bars represent 95% confidence intervals of the means of three independent replicates. The overlapping confidence intervals indicate no significant difference.

In order to confirm the starch phenotype of the experimental strains, wild-type (WT), complemented *STA6* mutant (*STA6-*C6) and starchless mutant (*sta6*) were spotted on plates with or without a nitrogen source, and subsequently stained with iodine vapour as described in the Materials and Methods section. As shown in Fig. 1a, we could observe a dark-brown colour in WT and *STA6-*C6 colonies under N-depleted conditions, whereas *sta6* colonies exhibited an opaque yellowish green colour indicating that starch could not be produced in this mutant. Additionally, the levels of lipid droplet accumulation in WT, *STA6-*C6 and *sta6* cells under N-depletion was also compared (Fig. 1b,c) to verify the ability to accumulate high amount of TAG of the *sta6* mutant as described previously^10,20^. As shown in Fig. 1b, the number of Nile Red-stained lipid droplets in the *sta6* mutant was more pronounced compared to WT and *STA6-*C6 mutant at 24 h after nitrogen starvation. In addition, fluorescence-based microplate assays revealed an approximate 5-fold and 8-fold increase in Nile Red fluorescent intensity in *sta6* cells at 24 h and 48 h post-starvation, respectively. Neutral lipid content of the control cells was also changed in response to nitrogen starvation but less significant compared to that observed in the *sta6* mutant.

### Change of daily growth and stress responses

**Figure 2:**
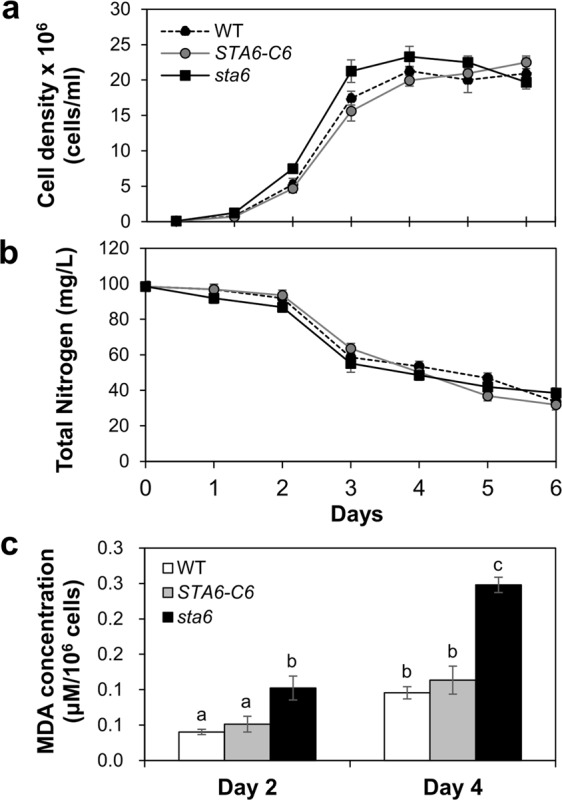
Comparison of growth rate and stress responses in *Chlamydomonas* strains. (**a**) Wild-type (WT), complemented *STA6* mutant (*STA6-*C6) and starchless mutant (*sta6*) exhibited similar growth patterns under optimal growth conditions. (**b**) Total nitrogen content in the medium gradually decreased correlating to algal growths. (**c**) The MDA contents were measured at day 2 (exponential phase) and day 4 (stationary phase) under batch culture conditions. The *sta6* mutant showed significantly higher stress levels compared to WT and *STA6*-C6 cells at the same growth stage. Data represent the mean ± standard deviation (SD). Means with the same letter are not significantly different from each other (*P* > 0.05 by *t*-test using Excel 2016 program).

After confirmation of the *sta6* mutant, daily growth tests were performed in TAP medium. As shown in Fig. 2a, the growth curves based on algal cell concentration of WT, *STA6-*C6 and *sta6* mutant were exponential up to day 3 before reaching a stationary phase that was maintained with a cell density of approximately 2 × 10^7^ cells/ml. The total nitrogen concentration was rapidly undermined at day 3 of cultivation, then slightly reduced thereafter as the cells entered the stationary growth phase (Fig. 2b). Based on these results, we considered that limiting nitrogen in the medium might induce algal cells to enter stationary growth phase after day 3.

Previous studies have reported that microalgae cultivated under nitrogen limiting conditions increased cellular ROS levels along with enhanced lipid accumulation, and the elevated ROS may inhibit algal growth by causing cellular damage^12,21^. Therefore, we measured the production of malondialdehyde (MDA), which is considered a marker of lipid peroxidation caused by enhanced oxidative stress, in algal samples collected at day 2 and day 4. As shown in Fig. 2c, the MDA content of WT cells was increased at day 4 compared to that at day 2 of cultivation, indicating the occurrence of lipid peroxidation and oxidative stress in stationary-phase cells. Interestingly, the *sta6* mutant showed significantly higher MDA levels than WT and *STA6-*C6 cells at the same growth phase, and a high level of MDA was observed even under optimal growth conditions (day 2 sample). These results suggest that the *sta6* mutant was likely exposed to higher oxidative stress levels compared to control cells growing in the same conditions.

**Figure 3:**
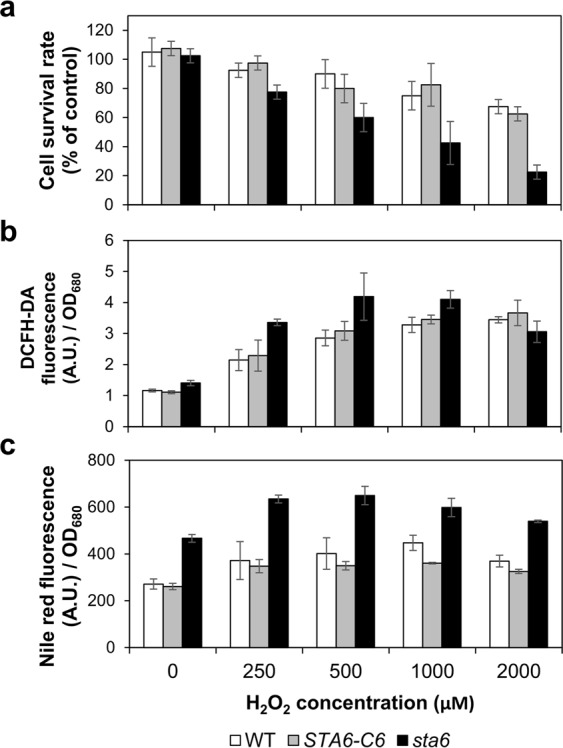
Effect of hydrogen peroxide (H_2_O_2_) on cell viability and lipid accumulation in *C. reinhardtii*. (**a**) Wild-type (WT), complemented *STA6* mutant (*STA6-*C6) and starchless mutant (*sta6*) were treated with H_2_O_2_ for 16 h and percentage of viable cells was determined. (**b**) Relative DCFH-DA fluorescence normalized to optical density (OD_680_) of algal cells after 6 h of H_2_O_2_ treatment. (**c**) Relative Nile Red fluorescence normalized to OD_680_ of algal cells after 16 h of H_2_O_2_ treatment. Error bars represent 95% confidence intervals of the means of three independent replicates. The overlapping confidence intervals indicate no significant difference.

Thus, as a further experiment, we compared the cellular sensitivity of WT, *STA6-*C6 and *sta6* to H_2_O_2_-induced oxidative stress conditions. Cells were grown to exponential phase and cell concentration was adjusted to 1 × 10^6^ cells/ml before treating with H_2_O_2_ to induce oxidative stress (Fig. 3). The concentrations of H_2_O_2_ higher than 250 μM rapidly triggered cell death in the *sta6* mutant within 16 h (Fig. 3a). Cell viability of the *sta6* mutant were 77.5 ± 3.5%, 60.0 ± 7.1%, 42.5 ± 10.6% and 22.5 ± 3.5% at 250, 500, 1,000 and 2,000 μM H_2_O_2_, respectively. In contrast, WT and *STA6-*C6 cells exhibited relatively higher resistance to H_2_O_2_-induced cell death (less than a 25% and 40% decrease in viability at 1,000 and 2,000 μM H_2_O_2_, respectively). These results indicate that the *sta6* mutant is more sensitive to oxidative stress compared to the WT and complemented *STA6* mutant, which is in accordance with the elevated cellular MDA content observed earlier.

### Effects of H_2_O_2_ on neutral lipid accumulation in *Chlamydomonas reinhardtii*

Treatment with H_2_O_2_ has been shown to effectively enhance lipid yield in *D. salina* and *Scenedesmus* sp.^12,13^. Indeed, the addition of H_2_O_2_ to the culture medium caused an increase in intracellular ROS levels in a wide range of organisms^22–24^. As cellular signalling molecules, the accumulated ROS may act as potent inducers of multiple metabolic functions including lipid metabolism and autophagy^25^. To determine whether the increased lipid accumulation in the *sta6* mutant correlates with its distinct oxidative profile, the cells in Fig. 3a were stained with DCFH-DA and fluorescence-based microplate was used to measure cellular ROS levels. The ROS contents were increased in a dose-dependent manner with H_2_O_2_ concentration in all strains (up to 500 μM H_2_O_2_ in case of *sta6* mutant) (Fig. 3b). At the concentrations of H_2_O_2_ higher than 500 μM, the *sta6* mutant had no increase in ROS levels but dramatic increase in cell death, indicating that *sta6* cells has reached an oxidative saturation point where the level of ROS exceeds cellular defence capacitance. Notably, we observed a correlation between increased neutral lipid content and increased cellular ROS levels in controls and *sta6* mutant (except for the 2,000 μM H_2_O_2_ condition). As shown in Fig. 3c, with WT cells exposed to increasing amounts of H_2_O_2_, lipid content increased from 271.2 ± 15.5 A.U. (non-treated control) to a maximum of 447.5 ± 23.2 A.U. (at 1,000 μM H_2_O_2_) (A.U. means arbitrary units). The changes in Nile Red fluorescence intensity for the *sta6* mutant were more pronounced compared to that observed in WT and *STA6-*C6 cells, which showed a positive, yet saturating relationship with the changes in its ROS levels (Fig. 3c). These results suggest that the overproduction of ROS in the *sta6* mutant may contribute to its elevated lipid accumulation.

### Differential autophagic activity in the *sta6* mutant

Autophagy is a catabolic process that usually occurs in eukaryotic organisms subjected to stress. The damaged cellular organelles caused by increased ROS are degraded and recycled via autophagic activity to maintain cellular homeostasis^26^. While the degradation of cellular materials is performed by the lysosome in animal cells, the same function is performed by lytic vacuoles in microalgae, fungi and plant cells^27^. As discussed in a previous review^28^, the autophagic machinery of *Chlamydomonas* comprises numerous autophagy-related (ATG) proteins. In the case of macroautophagy, hereafter referred to as autophagy, a double-membrane vesicle called the autophagosome is formed via a complicated mechanism. Autophagosome formation begins by the activation of the ATG1 complex composed of ATG1, ATG13 and probably ATG101^28,29^. ATG101, as previously described in the model terrestrial plant *Arabidopsis thaliana*, may help link the ATG1 initiation complex to autophagic membranes^30^. The phosphorylation of ATG1 triggers the formation of a phagophore assembly site (PAS) through phosphoinositide 3-kinase (PI3K) nucleation complex and phosphatidylinositol 3-phosphate (PI3P) binding complex. PI3K (VPS34) plays an important role in converting phosphatidylinositol (PI) to PI3P, which is required for the recruitment of other ATG proteins onto the autophagosome membrane. Among ATG proteins, ATG8 is essential for the formation of the autophagosome, while ATG7 is an E1-like enzyme involved in the activation of ATG8^28,30,31^.

**Figure 4:**
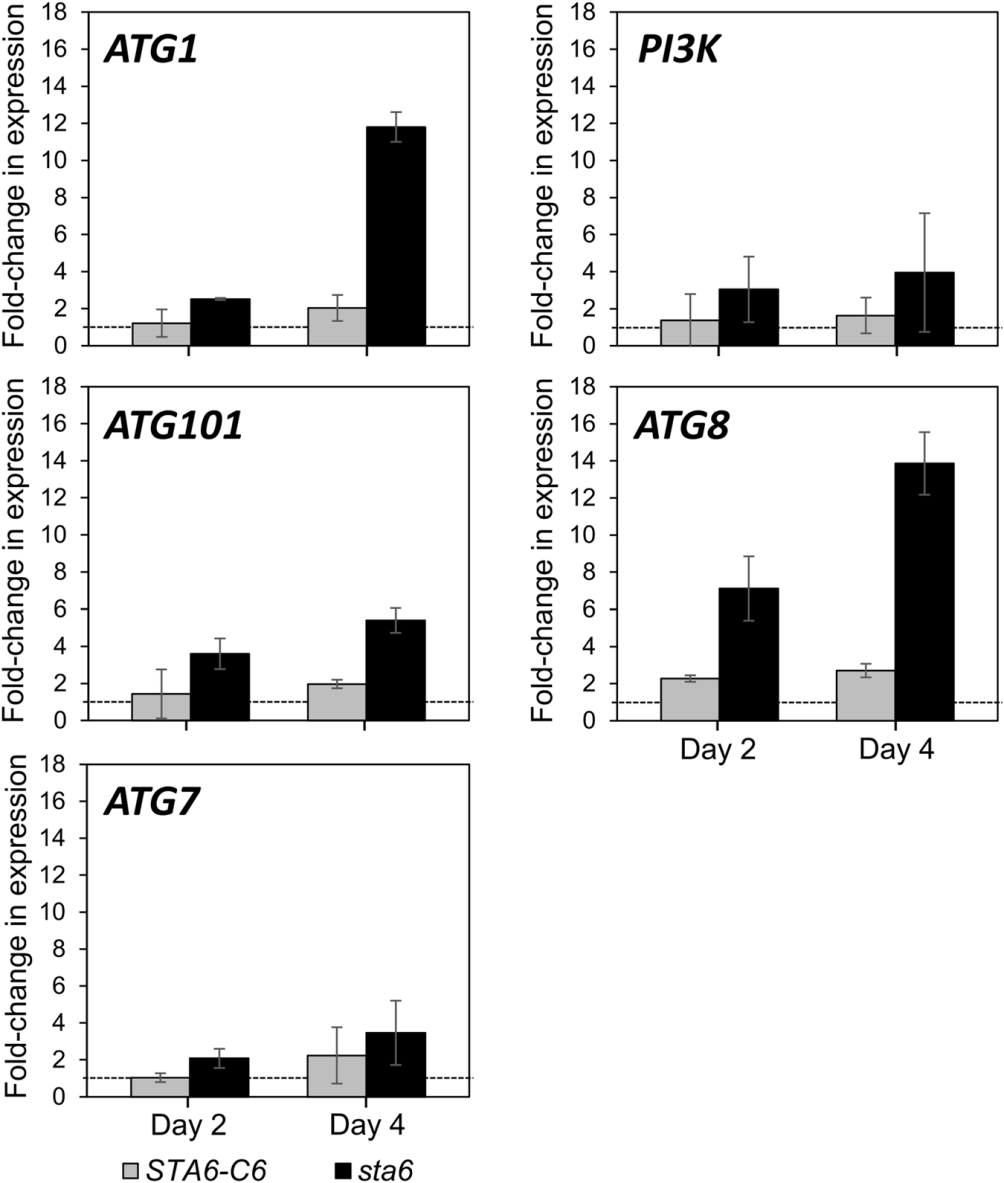
Expression levels of genes belonging to autophagy pathways. Transcript levels of autophagy-related (*ATG*) genes were measured by real-time qPCR in algal samples at day 2 (exponential phase) and day 4 (stationary phase). Relative expression levels of *ATG1*, *PI3K, ATG101*, *ATG8* and *ATG7* genes are shown. All results were normalized to *CBLP* (house-keeping gene). The bars represent the fold-change in expression of the target gene in *STA6*-C6 and *sta6* relative to wild-type. The dashed line indicates the expression level of corresponding genes in wild-type. Mean values of two biological replicates were obtained using the medians of three technical replicates. Error bars represent 95% confidence intervals. The overlapping confidence intervals indicate no significant difference.

In this study, we measured the expression levels of *ATG1*, *PI3K*, *ATG101*, *ATG7* and *ATG8* genes to evaluate autophagy regulation in the *sta6* mutant (Fig. 4). Compared to the expression levels of WT (marked as dash lines), all the autophagy-related genes in the *sta6* mutant showed higher values at both day 2 and day 4. Furthermore, the *ATG1* and *ATG101* genes, which regulate the early steps of the autophagic process, were significantly up-regulated in *sta6* cells after 4 days of cultivation, whereas *ATG7* and *PI3K* has shown no statistically significant changes. Moreover, the expression levels of the *ATG8* gene were significantly promoted in samples collected at D4 (when the cells reached stationary phase). Previous studies have demonstrated that increased cellular ROS may lead to the activation of *ATG1* and subsequent autophagy by either targeting rapamycin (TOR)-dependent or TOR-independent signalling^16^. Thus, the enhanced expression levels of those autophagy-related genes suggested that the *sta6* mutant activated autophagy mechanism to cope with its elevated oxidative stress levels.

**Figure 5:**
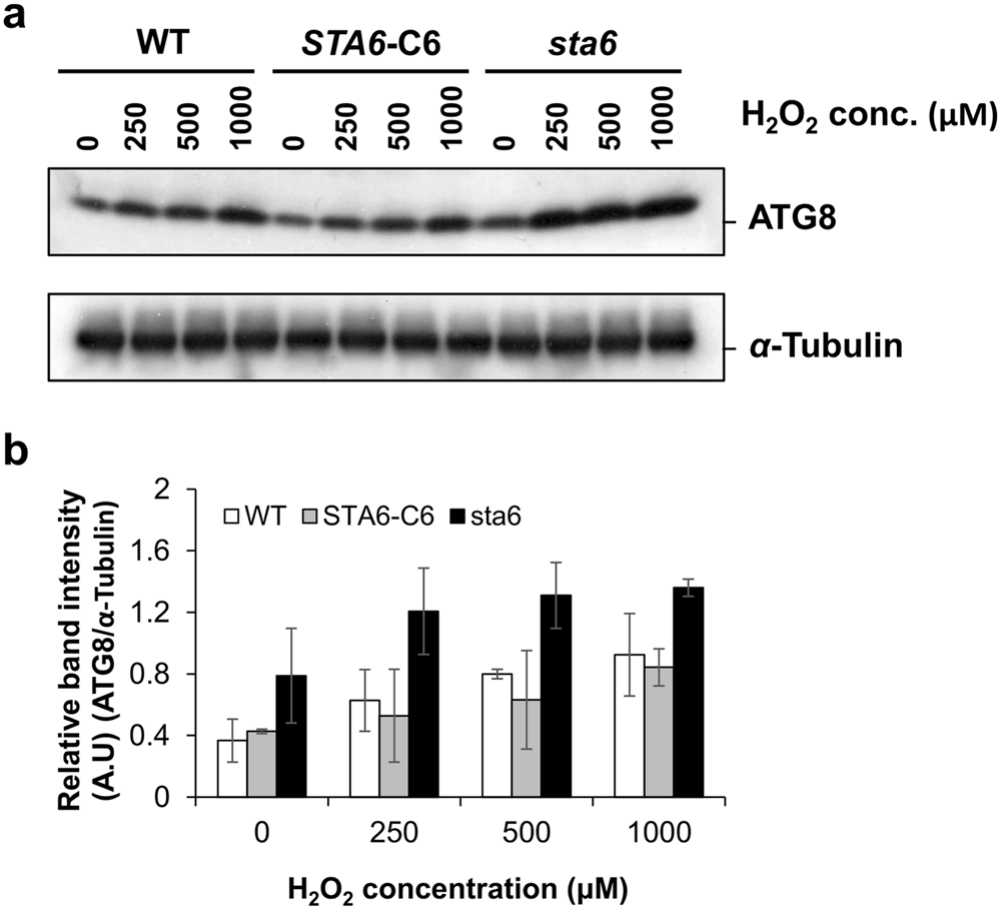
Differential autophagic responses in wild-type and starchless mutant. (**a**) Western blot analysis of ATG8 levels in wild-type (WT), complemented *STA6* mutant (*STA6-*C6) and starchless mutant (*sta6*) treated with H_2_O_2_ at concentrations of 250, 500, 1000 µM. *α*-tubulin is shown as a loading control. Although cropped, all blots are derived from the same gel. After transfer, the membrane was cut horizontally in half. The top half of the membrane was used to detect *α*-tubulin (molecular weight of approx. 50 kDa) and the bottom half was used to detect ATG8 protein (molecular weight of approx. 15 kDa). Corresponding uncropped blots with multiple exposures shown in Supplementary Figs S1 and S2. (**b**) Quantification of band intensities in (**a**) was performed using ImageJ. The band intensity of ATG8 was normalized to the according *α*-Tubulin and the ATG8/*α*-Tubulin ratios were displayed. Error bars represent 95% confidence intervals of the means of three independent replicates. The overlapping confidence intervals indicate no significant difference.

In addition, Western blot analysis was performed to examine the expression pattern of ATG8 protein in the *sta6* mutant. ATG8 appeared as a faint band on the blot of WT and *STA6-*C6 cells grown under optimal conditions (Fig. 5a, 0 μM H_2_O_2_). The abundance of ATG8 increased upon exposure to H_2_O_2_-induced autophagy in both controls and the *sta6* mutant (Fig. 5a). The quantification of protein band intensity was performed using ImageJ, claiming a significant increase of ATG8 protein in the *sta6* cells compared with WT or *STA6-*C6 mutant subjected to the same amount of stressor (Fig. 5b). Moreover, the dose-dependent expression pattern of ATG8 protein, observed in WT and *STA6-*C6 cells, was less significant in the *sta6* mutant (Fig. 5a,b). It seems that oxidative stress induces autophagy more strongly in the *sta6* mutant compared with WT and *STA6-*C6 mutant.

Based on these results, we propose that the high lipid accumulation in the *sta6* mutant could be explained from the perspective of stress responses and autophagy regulation, alongside carbon allocation as described previously. Although the underlying mechanism remains to be clarified, we speculate that the absence of starch biosynthesis likely led to increased levels of ROS and oxidative stress in the *sta6* mutant. Subsequently, the increased cellular ROS levels could act as autophagy inducers, leading to an enhanced recycling of cellular materials and ROS-damaged components to fuel lipid production in this mutant.

It has been reported that corn, which contains high levels of amylose, one of the two components of starch, has a good antioxidant capacity^32^. Also, largemouth bass *Micropterus salmoides* fed with pea starch and high-amylose maize starch showed a lower MDA content and reduced oxidative stress levels^33^. Similarly, oxidative stress suppression by resistant starch diet was also reported by Vaziri *et al*.^34^. In addition, starch biosynthesis has been shown to be an essential protection mechanism against photooxidative damage in *C. reinhardtii*^35^. The continuous generation of the reductant NADPH as an end product of the light reactions of photosynthesis leads to cellular over-reduction and the formation of ROS by transferring one electron from NADPH to oxygen^36^. In photosynthetic cells, ATP and NADPH produced by the light reactions are used to form sugars from carbon dioxide (CO_2_) via the Calvin cycle, and these sugars are further condensed into insoluble starch which has a lower osmotic pressure^37^. By consuming excess NADPH, the production of starch allows the algae to maximize light harvesting while regenerating NADP^+^ from NADPH which can accept electrons and thus reduce the generation of ROS^35^. Since lipid biosynthesis is the second NADPH-consuming process, an increased rate of lipid production in the *sta6* mutant would allow the cells to lower the NADPH pool^38,39^. In fact, the levels of oxidative damage in the *sta6* mutant were still significantly higher than that in the WT cells despite its elevated lipid accumulation. Juergens *et al*. highlighted that in *C. reinhardtii* starch is primarily produced from CO_2_ via photosynthesis, whereas the synthesis of fatty acids mostly relies on exogenous acetate and the turnover of membrane lipids^40^. Hence, this indicated that starch plays a more important role in the protection of the photosynthetic machinery from photooxidative damage than other bioproducts, including lipids and proteins^35,40^. Taken together, this suggests that one possible reason for the increase in lipid peroxidation in the *sta6* mutant may be the unbalanced redox state of the cells, leading to an elevated generation of ROS due to the lack of the starch biosynthesis pathway.

## Conclusion

Superior lipid accumulation in starchless mutants has been generally linked to increased carbon allocation towards the lipid metabolism in previous studies. This work has expanded on those findings from a different perspective, specifically focusing on the stress response and autophagy regulation. The increased lipid peroxidation observed in *sta6* cells growing under the same conditions as WT suggests that algal cells with impaired starch metabolism need to cope with elevated oxidative stress levels. The increased cellular ROS levels in *sta6* cells, therefore, could play a dual role as signals to trigger lipid biosynthesis and as autophagy inducers to activate the recycling of cellular components to fuel lipid production in this mutant. More research is needed to understand the antioxidant capability of purified algal starch molecules, and their roles in the regulation of related cellular pathways.

## Materials and Methods

### Microalgal growth conditions

*Chlamydomonas reinhardtii* CC-124 wild-type, complemented *STA6* mutant (referred to as *STA6-*C6) and starchless mutant BAFJ5 (referred to as *sta6*) were obtained from the *Chlamydomonas* Resource Center located at the University of Minnesota. In the *sta6* mutant, the small subunit of ADP-glucose pyrophosphorylase was deficient and the starch content was not detectable^41^.

Cells were grown in Tris-acetate phosphate (TAP) medium^42^ under continuous illumination of 50 ± 10 µmol/m^2^/sec at 25 °C, with constant shaking at 90 rpm. For growth rate comparison, cells were initially inoculated at a density of approximately 8 × 10^4^ cells/ml, and cell density was monitored daily using a haemocytometer.

For the nitrogen depletion studies, cells in exponential phase (at a density of approximately 1 × 10^6^ cells/ml) were harvested by centrifugation (2,000 × g for 5 min). The cell pellet was washed twice in nitrogen-free TAP medium before resuspension in the same fresh medium at the same cell density.

### Determination of algal starch content using iodine vapor staining

Pure elemental iodine crystals (Junsei Chemical Co., Ltd., Japan) were placed on a watch glass and heated slightly to generate iodine vapor inside a fume hood. To control the staining time, WT, *STA6-*C6 and *sta6* cells were spotted on the same plate. Agar plates containing algal colonies were placed on top of the watch glass for 30 s. Images were captured immediately after staining.

### Analysis of total nitrogen

Algal culture broth was filtered through a 0.2 µm pore-size membrane filter (Minisart, Sartorius Stedim Biotech, Germany) before measurement. The concentration of total nitrogen (TN) was determined using Nitrogen (total) Cell Test kit (Merck, Germany) in accordance with the manufacturer’s protocol.

### Determination of lipid peroxidation

Lipid peroxidation products are widely used to represent the levels of oxidative stress in cells. In this study, the thiobarbituric acid reactive substance (TBARS) assay was used to quantify the level of MDA content in microalgal cells (modified from^43^). Briefly, 1 ml samples with known number of cells were harvested by centrifugation at 2,000 × g for 5 min. Cell pellets were washed twice with cold phosphate buffered saline (PBS, pH 7.4) and resuspended in lysis buffer at a density of approximately 1 × 10^6^ cells/ml. Lysis buffer was prepared by mixing 1 µl of 10 mM butylated hydroxytoluene (BHT) solution per 100 µl of radio-immunoprecipitation assay (RIPA) buffer. The cell mixture was bead beaten at 4,500 × g for 1 min. Acid treatment was performed by adding 100 µl of 0.6 N trichloroacetic acid (TCA) solution per 100 µl of cell lysate and incubating at room temperature for 15 min. After centrifugation at 10,000 × g for 5 min, the supernatants were immediately assayed. The TBARS assay was performed by mixing 150 µl of each sample or MDA standard with 75 µl of 0.5% (w/v) thiobarbituric acid reagent. The optical density (OD) was determined at 532 nm immediately (pre-read OD_532_) and after incubating for 2 h at 45–50 °C. Pre-read OD_532_ was subtracted from the final read to obtain data, and MDA contents were calculated based on the MDA standard curve. The results are expressed as µM MDA/10^6^ cells.

### RNA extraction, cDNA synthesis, and real-time PCR

Cell culture was harvested by centrifugation at 2,000 × g for 5 min and the pellet was immediately frozen in liquid nitrogen and stored at −80 °C. Total RNA was extracted using RNeasy Mini Kit (QIAGEN, Germany) in accordance with the manufacturer’s protocol. To eliminate genomic DNA contamination, RQ1 RNase-Free DNase kit (Promega, United States) was used. RNA purity and quantity were checked with the NanoPhotometer^®^ P360 device (Implen, Germany), and RNA integrity was confirmed on agarose gel by electrophoresis. Then, 500 ng of total RNA was converted to first-strand cDNA in a 20 µl reaction using GoScript^TM^ Reverse Transcription System (Promega, United States) with oligo (dT) primer. The generated cDNA was diluted five times with RNase-Free water, and 1 µl of this solution was used as template for real-time PCR using iQ™ SYBR® Green Supermix (Biorad, United States). The following primers were used: ATG1_F (5′-GGGTGCGGTGGTGTACTAGC-3′) and ATG1_R (5′-AGTCCTCTTCCGGCACCGAT-3′); PI3K_F (5′-AGGAGATGATCGAGGCCATGGG-3′) and PI3K_R (5′-CCGGAACTTGTCCTGCAGCTTG-3′); ATG101_F (5′-CTTCTGTGCCCAGGTTGACAAGC-3′) and ATG101_R (5′-GCTCAACACCCACGTTTCCCAG-3′); ATG7_F (5′-GCATTGTAGGAGGGTGGTAGGAG-3′) and ATG7_R (5′-CTCCAGGTCTGTGTGGCTAGCTC-3′); ATG8_F (5′-CAGCATCTCCACAATGGTTGGC-3′) and ATG8_R (5′-CTCTGCCTTCTCGACAATGACTGG-3′).

### Western blot analysis

Cell lysates were prepared using RIPA buffer supplemented with Protease Inhibitor Cocktail (Sigma-Aldrich, United States). Then, 30 µg of total soluble protein per sample were separated on 15% SDS-PAGE gels and transferred onto a polyvinylidene difluoride membrane (Bio-Rad, United States) using a wet transfer system (Bio-Rad, United States). Western blot analysis was performed with anti-CrATG8 (Abcam, United Kingdom) and anti-*α*-tubulin (Sigma-Aldrich, United Kingdom) antibodies (1:2,000). The Clarity^TM^ Western ECL Substrate (Bio-Rad, United States) was used to detect ATG8 and *α*-tubulin with anti-rabbit (Abcam, United Kingdom) and anti-mouse (Abcam, United Kingdom) secondary antibodies (1:10,000), respectively.

### Algal lipid droplet observation by fluorescence microscope

*Chlamydomonas* cells were subjected to nitrogen starvation and microscopic observation was performed at 24 h and 48 h post-treatment. Lipid droplets were stained with Nile Red (Thermo Fisher, United States) and observed using a fluorescence microscope (Eclipse 80i, Nikon, Japan) at excitation 450–500 nm (blue laser) and emission 528 nm wavelength. Chlorophyll *a* (Chl *a*) auto-fluorescence was observed using the same filter.

### Fluorescence-based microplate assays for measurement of ROS levels and lipid contents

For quantification of cellular ROS levels, algal cells were treated with different H_2_O_2_ concentration and stained with dichloro-dihydro-fluorescein diacetate (DCFH-DA) (Sigma-Aldrich, United Kingdom). After cells were harvested and washed twice with PBS buffer (pH 7.0), DCFH-DA was added to final concentration of 10 µM and incubated for 30 min in the dark, at 25 °C, with constant shaking at 50 rpm. The DCFH-DA signals were then measured by a fluorescence microplate reader at excitation 485 nm and emission 535 nm wavelength.

For quantification of neutral lipid content, Nile Red was added to final concentration of 1 µg/ml and incubated for 30 min in the dark at 25 °C. The Nile Red signals were measured at excitation 560 nm and emission 635 nm wavelength. All experiments were performed in triplicates. Data were normalized against OD_680_ and the ratio of DCFH-DA fluorescence/OD_680_ or Nile Red fluorescence/OD_680_ are shown.

### Statistical analysis

All data are presented as means ± (1.96 × standard error (SE)) for a 95% confidence interval (unless otherwise indicated). The overlapping error bars indicate no significant difference. Pairwise comparisons of data from different experimental groups were performed using a *t*-test (Excel 2016 program, Microsoft). Statistically significant differences are represented by different letters (Fig. 2).

## Supplementary information

Supplementary Information
