# Supplementary material for: Impairment of starch biosynthesis results in elevated oxidative stress and autophagy activity in Chlamydomonas reinhardtii
Source: Sci Rep. 2019 Jul 8;9:9856. doi: 10.1038/s41598-019-46313-6 (PMC6614365; doi:10.1038/s41598-019-46313-6)
Supplement: Supplementary file 1 — Supplementary Information [file 41598_2019_46313_MOESM1_ESM.docx]

**Impairment of starch biosynthesis results in elevated oxidative stress and autophagy activity in *Chlamydomonas reinhardtii***

Quynh-Giao Tran^1,2,a^, Kichul Cho^1,a^, Su-Bin Park^1,2^, Urim Kim^1,2^, Yong Jae Lee^1^, Hee-Sik Kim^1,2*^

^1^Cell Factory Research Center, Korea Research Institute of Bioscience and Biotechnology (KRIBB), Dajeon 34141, Republic of Korea

^2^Department of Environmental Biotechnology, KRIBB school of Biotechnology, Korea University of Science & Technology (UST), Daejeon 34113, Republic of Korea

^a^These authors are contributed equally to this work

**^*^Corresponding author:** Dr. Hee-Sik Kim, Cell Factory Research Center, Korea Research Institute of Bioscience and Biotechnology (KRIBB), Daejeon 34141, Republic of Korea

Tel. +82-42-860-4326; Fax. +82-42-860-4594; E-mail: [hkim@kribb.re.kr](mailto:hkim@kribb.re.kr)

**Supplementary Figure S1**

**
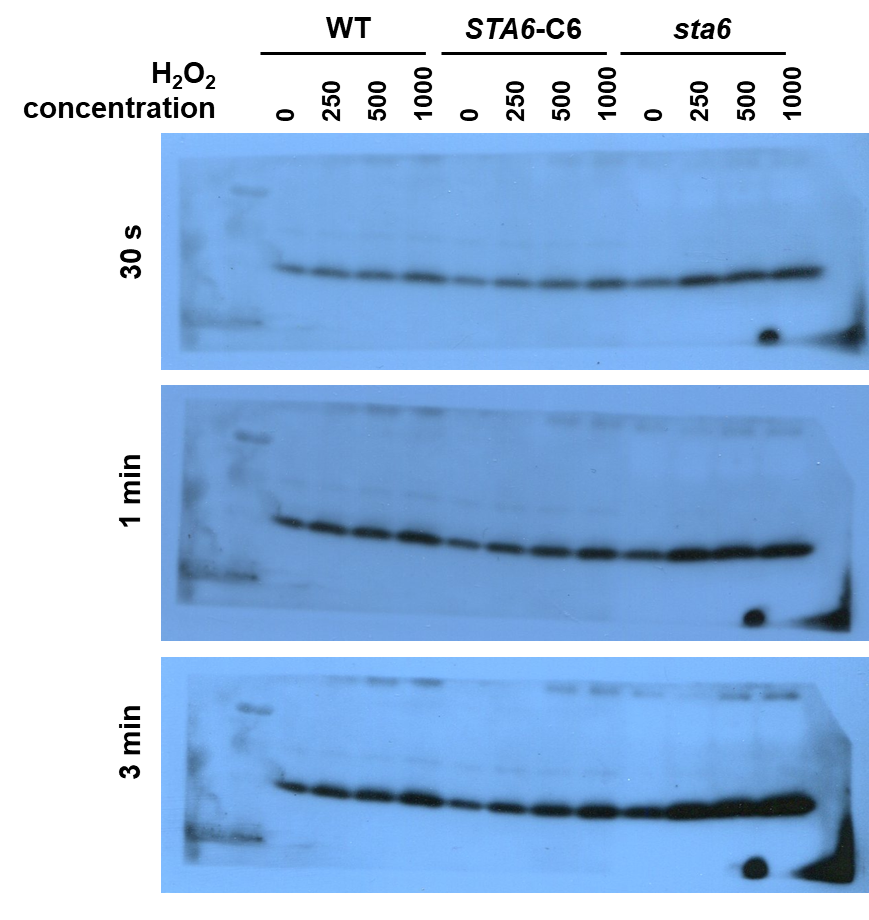
**

**Supplementary Figure S1. Uncropped blots/gels for Fig. 5a-ATG8 protein.** After transfer, the membrane was cut horizontally, and the bottom half was used to detect ATG8 protein (molecular weight of approx. 15 kDa). Multiple film exposures are captured from the same blot at different time points (exposure time indicated on the left side of each piece of film). The developed films were then scanned simultaneously at high resolution by a scanner (Epson Perfection V30) to obtain a digital image.

**Supplementary Figure S2**

**
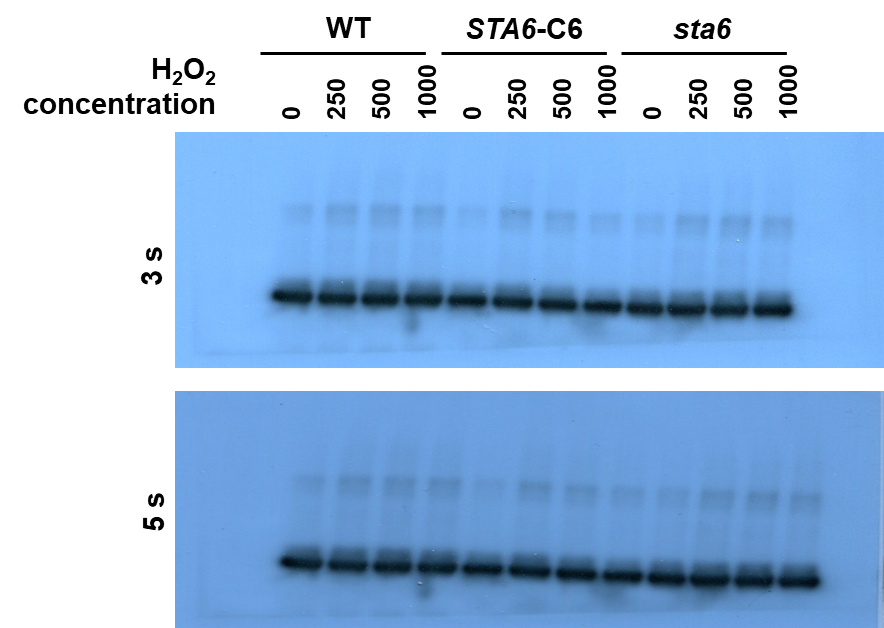
**

**Supplementary Figure S2. Uncropped blots/gels for Fig. 5a-*α*-tubulin protein.** After transfer, the membrane was cut horizontally, and the top half was used to detect *α*-tubulin (molecular weight of approx. 50 kDa). Multiple film exposures are captured from the same blot at different time points (exposure time indicated on the left side of each piece of film). The developed films were then scanned simultaneously at high resolution by a scanner (Epson Perfection V30) to obtain a digital image.
